# Supplementary material for: A Cinnamate 4-HYDROXYLASE1 from Safflower Promotes Flavonoids Accumulation and Stimulates Antioxidant Defense System in Arabidopsis
Source: Int J Mol Sci. 2023 Mar 11;24(6):5393. doi: 10.3390/ijms24065393 (PMC10049626; doi:10.3390/ijms24065393)
Supplement: Supplementary file 1 [file ijms-24-05393-s001.zip › ijms-2217887-supplementary.pdf]

Supplementary Figures and Tables

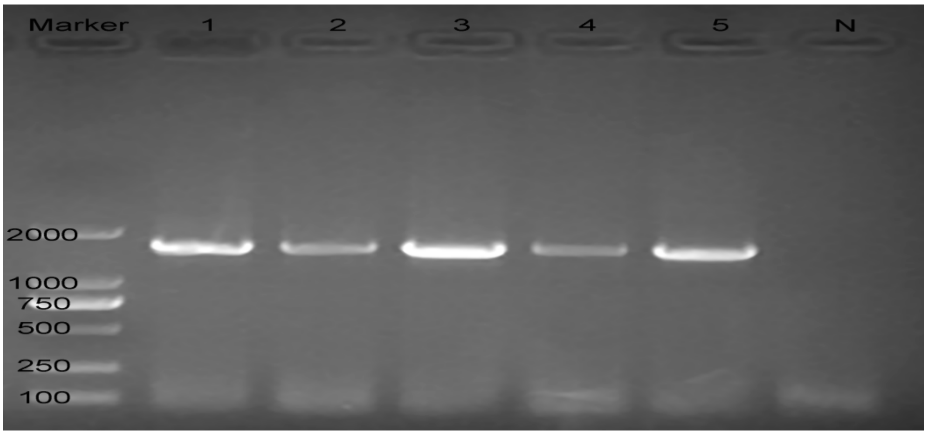

Figure S1. The PCR detection results of CtC41 gene cloning.

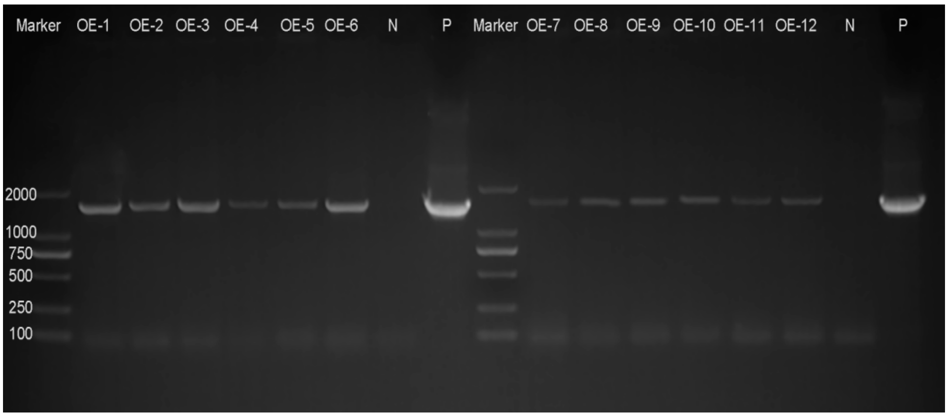

Figure S2. The PCR detection results of overexpressed lines.

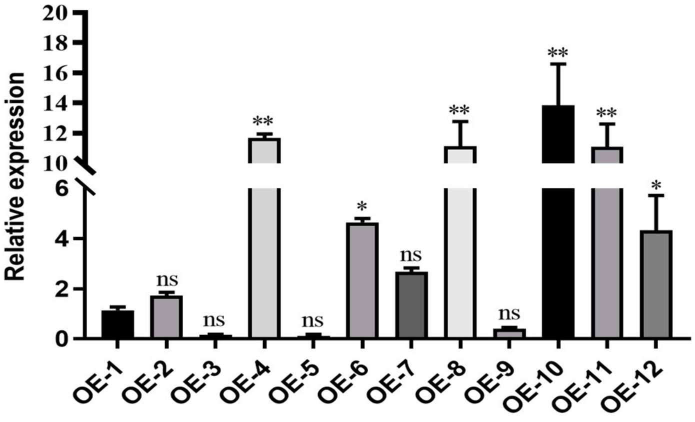

Figure S3. The qRT- PCR results of two high overexpressed lines.

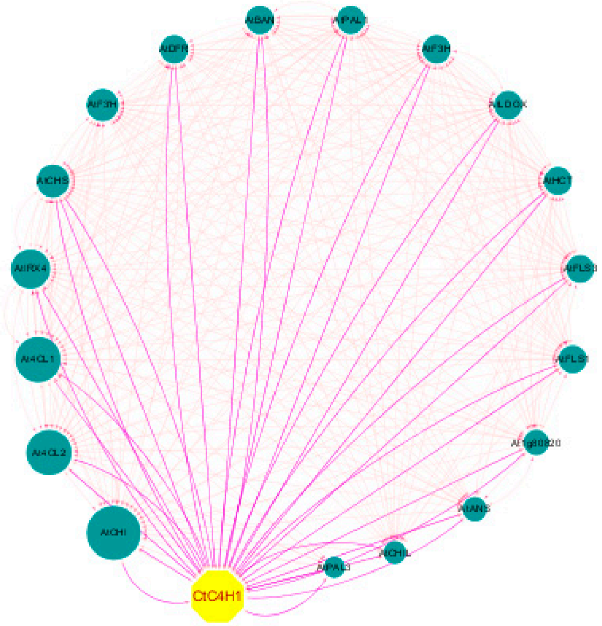

Figure S4. C4H Protein interaction network prediction

Supplementary Table S1

Cynara cardunculus	Cc
Zinnia elegans	Ze
Vigna radiata	Vr
Verbena x hybrida	Vh
Vaccinium corymbosum	Vc
Tripterygium wilfordii	Tw
Tarenaya hassleriana	Th
Tanacetum cinerariifolium	Tc
Salvia splendens	Ss
Sesamum indicum	Si
Rubus sp	Ru
Rubus occidentalis	Ro
Rubus coreanus	Rc
Pyrus sinkiangensis	Ps
Platycodon grandiflorus	Pg
Perilla frutescens	Pf
Pyrus x bretschneideri	Pb
Prosopis alba	Pa
Ocimum tenuiflorum	Ot
Osmanthus fragrans	Of
Olea europaea var	Oe
Ocimum basilicum	Ob
Nelumbo nucifera	Nn
Malus hybrid cultivar Royalty	MR
Mentha x piperita	Mp
Malus domestica	Md
Momordica charantia	Mc
Lactuca sativa	Ls
Lycoris radiata	Lr
Leucaena leucocephala	Ll
Lotus japonicus	Lj
Juglans regia	Jr
Isatis tinctoria	It
Ipomoea purpurea	Ip
Ipomoea nil	In
Ipomoea batatas cultivar purple	Ib
Hibiscus syriacus	Hs
Hibiscus cannabinus	Hc
Helianthus annuus	Ha
Fragaria vesca subsp	Fv
Echinacea purpurea	Ep
Euphorbia maculata	Em
Erythranthe guttatus	Eg
Erigeron canadensis	Ec
Echinacea angustifolia	Ea
Camellia sinensis	Cs

Cucurbita pepo subsp	Cp
Cucurbita moschata	Cm
Cirsium japonicum	Cj
Cichorium intybus	Ci
Citrus clementina	Cc
Chrysanthemum boreale	Cb
Barnadesia spinosa	Bs
Brassica rapa subsp	Br
Aquilaria sinensis	As
Agastache rugosa	Ar
Abrus precatorius	Ap
Asparagus officinalis	Ao
Arachis ipaensis	Ai
Arachis hypogaea	Ah
Arachis duranensis	Ad

Supplementary Table S2. Primers used in the paper.

Primer	Sequence (5'-3')
CtC4H1-F	ATGGATCTTCTCCTCTTGGAG
CtC4H1-R	TCACAACGATCTTGGTTTCGC
pCAMBIA1300-CtC4H1-F	ggacgagctcggtacccgggATGGATCTTCTCCTCTTGG
pCAMBIA1300-CtC4H1-R	ccatgtcgactctagaggatcCAACGATCTTGGTTTCGC
pGBKT7-CtC4H1-F	atggccatggaggccgaattcATGGATCTTCTCCTCTTGG
pGBKT7-CtC4H1-R	ccgctgcaggtcgacggatccCAACGATCTTGGTTTCGC
Pxy106-CtC4H1-F	atcgaggacgccggcggatccATGGATCTTCTCCTCTTGG
Pxy106-CtC4H1-R	acgaaagctctgcaggtcgacCAACGATCTTGGTTTCGC
pCAMBIA1300-CtPAL1-F	ggacgagctcggtacccgggATGGATCAATACATGAGCAATGGAC
pCAMBIA1300-CtPAL1-R	ccatgtcgactctagaggatcTGAAGAAATAGGAAGTGGGGTCCCA
pGADT7-CtPAL1-F	tatggccatggaggccagtgATGGATCAATACATGAGCAATGGAC
pGADT7-CtPAL1-R	tacgggtgggcccaccttaaTGAAGAAATAGGAAGTGGGGTCC
pxy104-CtPAL1-F	ttacaattacaggtacccgggATGGATCAATACATGAGC
pxy104-CtPAL1-R	gccaccgccgtcgactctagaTGAAGAAATAGGAAGTGG

Supplementary Table S3. Primers sequence used for qRT-PCR (5’-3’).

Primer	Sequence (5’-3’)
qPCR-CtC4H1-F	GCGTTTGGTGCAGAATTTCG
qPCR-CtC4H1-R	TCGCAACAATGGTGGAATGG
qPCR-CtC4H2-F	TGGTGCAGAATTTCGAGCTG
qPCR-CtC4H2-R	AACGACCGTGGTTTAGCAAC
18srRNA-F	GAGAAACGGCTACCACATCCAA
18srRNA-R	TCGTTTGAGCCCGGTATTGTTA
qRT-AtPAL-F	GCAAATCCTTTTCGCAGAAGC
qRT-AtPAL-R	GTCGACCGTCAAGAATGTGGTC
qRT-At4CL-F	CGCAAACCCTTTCTTCACTC
qRT-At4CL-R	ACTCCGTCGTCGTTTTGAAG
qRT-AtCHS-F	AGAAGTTCAAGCGCATGTGC
qRT-AtCHS-R	AGAGAAGGAGCCATGTAAGCAC
qRT-AtCHI-F	CGGTATGCAACATGCCGAA
qRT-AtCHI-R	AAGATACTTGGCAATGGTTGCG
qRT-AtF3H-F	TCAGATCGTTGAGGCTTGTG
qRT-AtF3H-R	ATGTCGAAACGGAGCTTGTC
qRT-AtF3’H-F	GAGGAGCGTGACCACAA
qRT-AtF3’H-R	GACTGAGCTAGCCGGAGAGTC
qRT-AtFLS-F	TGCAGTGCATGTGAAGAAGC
qRT-AtFLS-R	CGAGACCTTCTTTCAACGCATC
qRT-AtDFR-F	ATTTGCCAAACGCCAAGACG
qRT-AtDFR-R	TGTTGCCACGTGGAAAACAC
qRT-AtANS-F	TGCAAACGATCAAGCCACTG
qRT-AtANS-R	TTGTCCACTCGCGTTGTTAG

Supplementary Table S4. Go enrichment of differentially expressed genes identified from safflower.

Category	GOID	Go term	pvalue	padj
BP	GO:0065007	Biological regulation	1.75493E-05	0.001175803
BP	GO:0071840	cellular component organization or biogenesis	8.60675E-05	0.003844349
BP	GO:0009987	cellular process	0.000137249	0.034979675
BP	GO:0032502	developmental process	0.000178014	0.034979675
BP	GO:0051179	localization	4.10665E-05	0.015257606
BP	GO:0008152	metabolic process	8.90522E-05	0.015257606
BP	GO:0032501	multicellular organismal process	8.90522E-05	0.015257606
BP	GO:0050896	Responses to stimuli	0.000179397	0.022071832
BP	GO:0048511	rhythmic process	0.000214707	0.022071832
BP	GO:0023052	signaling	0.00010446	0.035934288
CC	GO:0005623	cell	0.037581225	0.037678498
CC	GO:0034330	cell junction	0.03789663	0.011997768
CC	GO:0005576	extracellular region	0.03789663	0.067849842
CC	GO:0016020	membrane	0.04947279	0.01776785
CC	GO:0044425	membrane part	0.049912644	0.049776785
CC	GO:0031974	membrane-enclosed lumen	0.049912644	0.027856072
CC	GO:0043226	organelle	0.000543309	0.010269344
CC	GO:0044422	organelle part	0.000805316	0.051850717
CC	GO:0065003	protein-containing complex	0.001039723	0.030265212
CC	GO:0099080	supramolecular complex	0.003611977	0.040123684
CC	GO:0055044	symplast	0.000417837	0.02043221
CC	GO:0019012	virion	0.004675681	0.040150883
CC	GO:0044423	virion part	0.009816576	0.029977678
MF	GO:0016209	antioxidant activity	0.008432368	0.010491599
MF	GO:0005488	binding	0.00061913	0.002419217
MF	GO:0003824	catalytic activity	8.82592E-05	0.020559605
MF	GO:0098772	molecular function regulator	0.00778394	0.021275246
MF	GO:0060089	molecular transducer activity	8.39399E-05	0.044259895
MF	GO:0005198	structural molecule activity	0.003939939	0.898062569
MF	GO:0140110	transcription regulator activity	0.02994321	0.047703835
MF	GO:0005215	transporter activity	0.026568319	0.002289936
